# Calcium and magnesium concentrations in uterine fluid and blood serum during the estrous cycle in the bovine

**Published:** 2012

**Authors:** Sayed Mortaza Alavi-Shoushtari, Siamak Asri-Rezaie, Roya Abedizadeh, Amir Khaki, Mozhgan Pak, Sajad Alizadeh

**Affiliations:** *Department of Clinical Sciences, Faculty of Veterinary Medicine, Urmia University, Urmia, Iran.*

**Keywords:** Calcium, Magnesium, Bovine, Uterine fluid, Estrous cycle

## Abstract

To investigate uterine and serum Ca^++^ and Mg^++^ variations during the estrous cycle in the bovine, 66 genital tracts and blood samples were collected from Urmia abattoir, Urmia, Iran. The phase of the estrous cycle was determined by examination of the structures present on ovaries and uterine tonicity. Of the collected samples, 17 were pro-estrus, 12 estrus, 14 metestrus and 23 diestrus. The uterine fluid was collected by gentle scraping of the uterine mucosa with a curette. The mean ± SEM concentration of serum Ca^++^ in pro-estrus, estrus, metestrus and diestrus was 5.77 ± 0.69, 8.87 ± 1.83, 10.95 ± 1.52, 11.09 ± 1.08 mg dL^-1^, and the mean concentration of uterine fluid Ca^++^ was 4.40 ± 0.72, 3.15 ± 0.67, 5.89 ± 0.88, 8.63 ± 0.97 mg dL^-1^, respectively. The mean concentration of serum Mg^++^ in pro-estrus, estrus, metestrus and diestrus was 3.53 ± 0.30, 4.20 ± 0.52, 3.49 ± 0.38, 3.39 ± 0.29 mg dL^-1^, and mean concentration of uterine fluid Mg^++^ was 5.27 ± 0.42, 4.92 ± 0.60, 5.56 ± 0.30, 5.88 ± 0.36 mg dL^-1^, respectively. The serum and uterine fluid Ca^++^ in pro-estrus were significantly different from those of the metestrus and diestrus. In all stages of estrous cycle the mean concentration of serum Ca^++^ was higher than that in the uterine fluid. The difference between serum and uterine fluid Ca^++^ in estrus, metestrus and diestrus was significant. There was no significant difference between serum Mg^++^ content nor was it different from uterine fluid Mg^++^ content at any stages of estrous cycle. In all stages of estrous cycle the uterine fluid Mg^++^ was higher than that of the serum. These results suggest that during the estrous cycle in the cow, Ca^++^ is passively secreted in uterine fluids and is mostly dependent on blood serum Ca^++^ variations but Mg^++^ is secreted independently and does not follow variations in the serum concentrations.

## Introduction

Physiologically, calcium (Ca^++^) is classified as either intracellular or extracellular ion. The skeleton is a major reservoir for providing Ca^++^ for both the extra- and intracellular pools. The role of calcium in the cell functions, including spermatozoa has been reported.^1^^-^^7^ Magnesium (Mg^++^) is the second most prevalent intracellular cation and is involved in the metabolic activity of the cell.^3^ Intracellular Mg is involved in the activity of hormone receptor complex in the cell membrane.^6^

Ionic composition of the uterine and oviduct fluids has been shown to be important in the context of oocyte and spermatozoon maturation, fertilization and early embryo development.^8^ Ions, including Ca^++^ and Mg^++^, play an important role in the formation of oviduct and uterine fluid. They move through the epithelial cells of the uterus into the lumen of the reproductive tract causing a concentration gradient which in turn causes an osmotic gradient providing the driving force to transport water by osmosis out of the epithelial cells into the uterine lumen. Leese points out that ion concentration and their movement are essential for the regulation of enzyme activity and of the pH of the uterine fluid.^9^ The ionic composition of uterine fluid is apparently derived from a combination of ions from the blood and ions secreted from uterine epithelium.^8^

Endometrium has different cell structure which secrete a variety of different materials into the uterine lumen.^10^ Cyclicity has an important role in protection of reproductive tract against infections. This protection in the uterus is created by several mechanisms, for instance uterine motility in estrus in which the defense mechanisms are enhanced by plasma estradiol concentration, which is more than that in diestrus,^11^^,^^12^ helps in physical clearance of infectious agents.^13^^,^^14^ Secretion of immunoglobulins in the uterine lumen varies during the phases of the estrous cycle.^15^^-^^17^ Oviduct and uterine secretions are necessary for the spermatozoa capacitation and oocyte maturation^18^ and for the zygote survival.^19^^-^^21^ This is an important factor in preparing medium for *in vitro* fertilization and embryo culture.

Despite the importance of ions, including Ca^++^ and Mg^++^ in uterine fluid formation^22^ and in gamete motility,^23^^-^^25^ zygote and early embryo development^26^ the literature is poor on the Ca^++^ and Mg^++^ concentrations in the uterine fluid during the estrous cycle in cattle. This work was carried out to investigate Ca^++^ and Mg^++^ concentration variations in the uterine fluid and to compare them with concentrations in blood serum during the estrous cycle in bovine to find any possible inter-relationship.

## Materials and Methods

Genital tract and blood sample of 232 slaughtered cows with unknown history of reproduction and plain of nutrition were collected in Urmia abattoir, Urmia, Iran (37° 33΄ N, 45° 4΄ E) from November 2010 to June 2011. Samples were immediately transferred to the lab in a cold box. On an initial examination, all the immature and abnormal samples were discarded. Genital tracts were examined to determine the stage of their cycles by examining the structures on their ovaries and their uterine tonicity, as described by Noakes.^27^ Hence, presence of a matured CL and flaccid uterine horns were considered as diestrus, and presence of a large follicle and turgid uterine horns as estrus. Of 132 normal cyclic genital tracts, 20 pro-estrus, 13 estrus, 19 metestrus and 80 diestrus cases were selected for further examinations. Of these groups, 17 pro-estrus, 12 estrus, 14 metestrus and 23 diestrus samples with clear non-hemolyzed serum samples were finally used. Blood samples were collected through jugular vein puncture in plain glass tubes before slaughter and allowed to clot. Serum samples were harvested by centrifuging the clotted blood at 3000 rpm for 10 minutes, and stored in Eppendorf microtubes at -20 °C until examination. Hemolyzed blood samples were discarded. Uterine fluid samples were collected by gentle scraping of the mucosa using a curette after incision of uterine horns and stored in Eppendorf microtubes at -20 °C until examination. Uterine fluid and blood serum Ca^++^ and Mg^++^ contents of the samples were determined by a spectrophotometry method using commercial kits (Ca+ and Mg^++^ assay kit, Elitech, France) after thawing the samples. 

The data were analyzed using SPSS software Version 18 (SPSS Inc., Chicago, IL, USA) computer program. Data were analyzed by one way ANOVA, statistic mean and standard error of mean were calculated for each group, the groups were compared by paired Student’s *t-*test, and the significance was set at *P* < 0.05. 

## Results

The mean ± SEM Ca^++^ value obtained for all the 66 blood serum and uterine fluid samples were 9.50 ± 0.67 mg dL^-1^ and 5.96 ± 0.51 mg dL^-1^, respectively, which were significantly different (*P* < 0.01) (Table 1). Serum Ca^++^ concentration in pro-estrus (5.77 ± 0.69 mg dL^-1^) was significantly different from those in metestrus (10.95 ± 1.52 mg dL^-1^) (*P* < 0.01) and diestrus (11.09 ± 1.08 mg dL^-1^) (*P* < 0.01).The mean Mg^++^ value for all serum samples was 3.59 ± 0.17 mg dL^-1^, while the value of 5.48 ± 0.21 mg dL^-1^ was obtained for all uterine fluid samples, which were significantly (*P* < 0.01) different (Table 1).

The mean uterine fluid Ca^++^ concentration in diestrus (8.63 ± 0.97 mg dL^-1^) was different (*P* < 0.01) from those of the other phases (Table 2). 

**Table 1:** Serum and uterine fluid samples total Ca^++^ and Mg^++^ concentrations (Mean ± SEM).

Parameters	No.	Serum	Uterine fluid	*P* value
Ca^++^ (mg dL^-1^)	66	9.50 ± 0.67*	5.96 ± 0.51	0.000
Mg^++^ (mg dL^-1^)	66	3.59 ± 0.17*	5.48 ± 0.21	0.000

* denotes difference between rows at *P* < 0.01 level.

**Table 2:** Serum and uterine fluid Ca^++^ concentrations in different phases of estrous cycle of the cow (Mean ± SEM).

Phases	No.	Serum (mg dL^-1^)	Uterine fluid (mg dL^-1^)
Pro-estrus	17	5.77 ± 0.69^a^	4.40 ± 0.72^a^
Estrus	12	8.87 ± 1.83*	3.15 ± 0.67^a^
Metestrus	14	10.95 ± 1.52**^b^	5.89 ± 0.88
Diestrus	23	11.09 ± 1.08**^c^	8.63 ± 0.97^b^

* and denote difference between rows at *P* < 0.05 and *P* < 0.01 levels, respectively; different letters (a, b, and c) denote a significant difference within columns.

The mean serum Mg^++^ concentration in pro-estrus was 3.53 ± 0.30 mg dL^-1^. It was 4.20 ± 0.52 mg dL^-1^ in estrus, 3.49 ± 0.38 mg dL^-1^ in metestrus and 3.39 ± 0.29 mg dL^-1^ in diestrus (Table 3). The mean Mg^++^ concentration in uterine fluid samples was 5.27 ± 0.42 mg dL^-1^ in pro-estrus. It was 4.92 ± 0.60 mg dL^-1^ in estrus, 5.56 ± 0.30 mg dL^-1^ in metestrus and 5.88 ± 0.36 mg dL^-1^ in diestrus. Serum Mg^++^ contents in different stages of the cycle were not significantly different nor were the uterine fluids (Table 3).

**Table 3:** Serum and uterine fluid Mg^++^ concentrations in different phases of estrous cycle of the cow (Mean ± SEM).

Phases	No.	Serum (mg dL^-1^)	Uterine fluid (mg dL^-1^)
Pro-estrus	17	3.53 ± 0.30*	5.27 ± 0.42
Estrus	12	4.20 ±0.52	4.92 ± 0.60
Metestrus	14	3.49 ± 0.38*	5.56 ± 0.30
Diestrus	23	3.39 ± 0.29*	5.88 ± 0.36

* denotes difference between rows at *P *< 0.01. No significant difference was observed within columns.

Serum Ca^++^ concentration in pro-estrus was significantly different from that of metestrus (10.95 ± 1.52 mg dL^-1^) (*P* = 0.006). Calcium content of the uterine fluid in diestrus (8.63 ± 0.97 mg dL^-1^) which was the highest Ca^++^ value in uterine fluid samples was significantly different from those of pro-estrus (4.4 ± 0.72 mg dL^-1^) (*P* = 0.003) and of estrus (3.15 ± 0.67 mg dL^-1^) (*P* = 0.000). It was noticed that in all stages of the cycle Ca^++^ concentrations were higher in the serum than in the uterine fluids, and in estrus (*P* = 0.011), metestrus, (*P* = 0.001) and diestrus (*P* = 0.004) a significant difference was observed (Table 2).

The highest serum Mg^++^ value (4.20 ± 0.52 mg dL^-1^) observed in estrus and the lowest (3.39 ± 0.29 mg dL^-1^) in diestrus were not significantly different(*P *> 0.05). Also, the highest Mg^++^ concentration in uterine fluid samples observed in diestrus (5.88 ± 0.36 mg dL^-1^) was not significantly different from the lowest value of estrus (4.92 ± 0.60 mg dL^-1^). The mean Mg^++^ concentrations of serum or uterine fluid samples in none of the stages were significantly different, but the values in uterine fluid samples were slightly higher than the serum samples. The difference of Mg^++^ contents of serum and uterine fluid samples in pro-estrus (*P* = 0.002) and metestrus (*P* = 0.002) was significant (Table 3).

## Discussion

During the first 20 days of pregnancy development of embryo is dependent on the nutrients provided by uterine fluid for its growth and survival.^8^ Despite the fact that more than 40% of the embryos in cattle are lost before their attachment to uterine wall, little information is published on concentration of Ca^++^ and Mg^++^ in the uterine fluid or on their variations during the stages of the estrous cycle. This led us to investigate the variation in concentrations of these ions at different stages of the estrous cycle in continuation of our previous works.^28^^,^^29^ Different methods of collecting uterine fluid samples have been used by other workers which had some problems such as condensation of the flushing or imposing a surgical operation on the animal. The method we used in this study had none of these problems and the secretions were directly analyzed. 

The mean Ca^++^ value obtained in this study for all the serum samples (9.50 ± 0.67 mg dL^-1^) was in agreement with the report of Hoffman who reported the mean normal value of 10.20 ± 0.28 mg 100mL^-1^ for bovine blood plasma calcium content;^30^ Blood and Radostits who reported 8.00 to 10.50 mg dL^-1^ in the bovine blood serum;^31^ and Radostits *et al*., who reported the mean normal value of bovine serum Ca^++^ ranging from 9.70 to12.40 mg dL^-1^.^32^ These authors point out that there may be variations in plasma Ca^++^ concentrations as a result of reproductive state, age or plane of nutrition of the animals. 

In this study the highest serum Ca^++^ was observed in diestrus (11.69 ± 1.08 mg dL^-1^) and the lowest (5.77 ± 0.69 mg dL^-1^) in pro-estrus which were significantly different. This was in agreement with the report of Jordan *et al*. that blood plasma Ca^++^ content increases with elevations in plasma progesterone concentration,^33^ but is contrary to the report of Hugentobler *et al*. who found no significant difference in bovine serum Ca^++^ at days 2 and 14 of the cycle.^8^ A significant difference of the serum Ca^++^ concentration in pro-estrus with that of metestrus observed here could be the consequences of the hormonal changes occur in these two stages of estrous cycle.

In this study the highest uterine fluid Ca^++^ content (8.63 ± 0.97 mg dL^-1^) was observed in diestrus and the least (3.15 ± 0.67 mg dL^-1^) in estrus, in which the difference was significant. It was also significantly different from that observed in pro-estrus. This was also in agreement with the report of Jordan *et al*.^33^ The least uterine fluid Ca^++^ and Mg^++^ contents observed in estrus might be caused by the dilution of the secretions which occur in this stage as a result of increased uterine blood flow induced by estradiol or could be due to the negative effect of the hormone. It was noticed that uterine fluid Ca^++^ content in all stages of cycle is lower than that of the serum and followed its changes which could be explained by its passive secretion in the uterine lumen. 

Serum Mg^++^ content observed in this study (3.59 ± 0.17 mg dL^-1^) was in agreement with the report of Hoffman who reported that the mean value of bovine blood plasma Mg^++^ was 2.89 ± 0.25 mg 100 mL^-1^;^30^ and Blood and Radostits who reported 1.20 to 3.50 mg dL^-1^ in bovine blood serum,^31^ but does not agree with that of Radostits *et al*., who reported the mean value of bovine serum Mg^++^ 1.80-2.30 mg dL^-1^.^32^

In this study the highest value for serum Mg^++^ concentration was observed in estrus (4.20 ± 0.52 mg dL^-1^) and the lowest in diestrus (3.39 ± 0.29 mg dL^-1^), but no significant difference was noted between the stages of the cycle. This is in agreement with the report of Hugentobler *et al*. who found no significant difference in bovine serum Mg^++^ during the stages of the cycle,^8^ however, contrary to their finding of the highest serum Mg^++^ value in diestrus, and to the report of Jordan *et al*. that blood plasma Mg^++^ content increased with elevations in plasma progesterone concentration.^33^ Our result of the highest uterine fluid Mg^++^ content in diestrus (5.88 ± 0.36 mg dL^-1^) and finding no significant difference between Mg^++^ content in different phases of the estrous cycle are in agreement with the reports of Hugentobler *et al*. who found higher uterine fluid Mg^++^ content at days 6 and 8 of the cycle than that of day 14,^8^ and with report of Jordan *et al*., who found that uterine fluid Mg^++^ content increased with a rise in serum progesterone concentrations.^33^ In this study the higher Mg^++^ content of uterine fluids in all stages of the cycle than those of the serum is in agreement with Hugentobler *et al*.^8^

The results of the present study indicated that calcium secretion in uterine fluid followed the changes which occurred in serum calcium content during the phases of the estrous cycle and it was usually lower than that of the serum values. In diestrus, in which the uterine blood flow was less than the other phases of the cycle, Ca^++^ level in the uterine secretions was the highest which may suggest an active secretion. But, Mg^++^ levels in the uterine fluid were higher than that in the serum and did not change with the variations in the serum values at different stages of the cycle, which suggested that its secretion in the uterine lumen was an active one. 
